# Pickleball- and Paddleball-Related Injuries to the Upper Extremity

**DOI:** 10.7759/cureus.39831

**Published:** 2023-06-01

**Authors:** Alexis A Kasper, John L Gibbons, Jack Abboudi, Daren Aita, T. Robert Takei, Daniel Fletcher, Greg G Gallant, Moody Kwok, Pedro Beredjiklian

**Affiliations:** 1 Division of Hand Surgery, Rothman Orthopaedic Institute, Philadelphia, USA; 2 Department of Orthopaedic Surgery, Thomas Jefferson University, Philadelphia, USA

**Keywords:** injury treatment, outpatient clinic, upper extremity, paddleball, pickleball

## Abstract

Introduction

While pickleball and paddleball are rapidly growing as popular sports in the United States, research on the incidence of hand and upper extremity injuries and treatments in outpatient clinics are lacking. This study evaluates the incidence rates and treatment options, both surgically and nonsurgically, for patients presenting with pickleball/paddleball-related injuries.

Methods

A retrospective database search of our multispecialty, multilocation electronic medical record (EMR) system from 2015 to 2022 identified 204 patients with outpatient pickleball- and paddleball-related injuries. The data from these patients’ charts were reviewed for injury incidences, treatment trends, and demographics.

Results

The majority of patients suffered wrist fractures due to a fall/dive and were treated nonsurgically. The most common surgical treatment, when required, was open reduction and internal fixation of the distal radius. We found that pickleball and paddleball players who sustained wrist fractures required surgery at a higher rate than the general population if above the age of 65.

Conclusion

As pickleball and paddleball continue to gain popularity, hand surgeons should be aware of the types of injuries that can occur and, when possible, counsel patients accordingly to try to prevent them. Additionally, hand surgeons should recognize the common treatments and outcomes that arise from pickleball/paddleball-related injuries.

## Introduction

Pickleball and paddleball are among the fastest-growing sports in the United States [1,2]. Although seemingly similar to tennis, both sports differ from tennis in multiple aspects, including the degree of running, court size, equipment size and weight, and scoring. Pickleball and paddleball are played on courts smaller than a tennis court: 44 ft x 20 ft and 34 or 40 ft x 22 ft, respectively, compared to tennis’s 78 ft x 36 ft. Additionally, paddleball is played in a cage, whereas pickleball is played on an open court. Paddleball allows the ball to be hit off the cage multiple times during play, whereas pickleball and tennis have a more straight-forward back-and-forth play [3,4]. 

There has been significant research into the risks and treatment options for common tennis injuries, but very few papers have been published regarding the risks and treatments of pickleball and paddleball injuries. The slight variations in the style of game create a different dynamic and risk of trauma between pickleball and paddleball compared to tennis. Traditionally, both pickleball and paddleball have been reserved for aging athletes, but the rising popularity of both games has sparked interest in players of all ages [1,5]. For the 65 and older population, pickleball has especially allowed for more physical activity in a group that is rising in numbers and age, which results in many health benefits [6]. The shift in demographics in the two similar sports has modified both the amount of and the types of injuries being presented to emergency departments (EDs) and outpatient clinics throughout the past years.

 Current research into pickleball and paddleball injuries is growing but incomplete. Prior papers have analyzed the most common emergency room pickleball injuries across the country; however, there are little data available regarding the treatments of individual patients [7,8]. With the increasing diversity of players (age, skill level, athletic fitness, etc.), it is valuable that hand/upper extremity surgeons become knowledgeable about the common risks and injuries involved with these increasingly popular new sports. The purpose of this study is to review pickleball- and paddleball-related injuries of the hand and upper extremity presented to orthopedic clinics in the Philadelphia area. We expect the trauma patterns to be similar between the orthopedic clinic and emergency rooms.

## Materials and methods

Thomas Jefferson University Institutional Review Board (IRB) approval (iRISID-2022-1035) and a waiver of informed consent per institutional protocol were obtained. Once IRB approval was obtained, a retrospective database search in our internal electronic medical record (EMR) system was conducted using the key terms "pickle," "paddle," "pickleball," and "paddleball" to establish all outpatient pickleball- and paddleball-related injuries. All related incidences were compiled from 2015 to 2022 for 20 fellowship-trained hand surgeons from a large, multispecialty orthopedic practice. The term "paddle tennis" was also included as another term for paddleball. After a chart review of the patients identified through the EMR search, 204 patients with 217 injuries met the inclusion criteria for pickleball-related and paddleball-related injuries.

The data collected during chart review included both demographical and clinical aspects of patients. The patient’s age, sex, anatomic location of injury, type of injury, mechanism of injury, timing between injury and initial office evaluation, and treatment course were compiled. Treatment was categorized as either nonoperative or surgical, and an effort was made to distinguish whether the injury was paddleball- or pickleball-related. For those treated surgically, the time from initial injury to surgical intervention was also noted. For those treated nonoperatively, conservative treatment(s) were recorded.

## Results

We identified 204 patients who presented for treatment for pickleball-related (171)/paddleball-related (33) injuries. This cohort included 137 women (67.2%) and 67 men (32.8%), with an average age of 65 (range 14-92). 56% of patients came in with a right upper extremity injury (n=115), while 38% came in with a left upper extremity injury (n=78). Eleven patients came in for bilateral injuries (6%). The average time from injury to first outpatient visit was 68 days (range of 0-1096 days). Out of the 204 patients, 39 (19.1%) had surgical treatment, while 165 (80.9%) were treated nonoperatively. Nonoperative treatments included steroid injections (26.1%), therapy (44.2%), and splinting/bracing (69.1%). Those treated surgically first in the office had an average of 55.8 days following the initial injury (range 0-731 days), while patients who had nonoperative treatment first presented an average of 72.2 days following the initial injury (range 0-1096 days). The average time to surgery from the initial injury was 88.5 days (range 1-1014 days).

**Figure 1:**
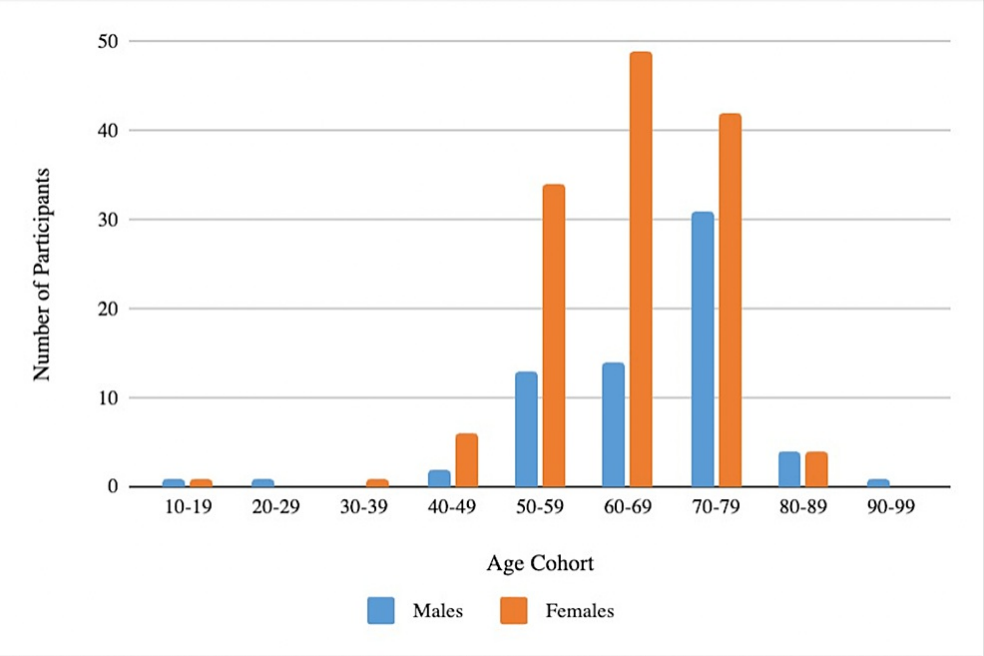
Age distribution of injuries based on sex.

Age distribution of upper extremity injuries based on sex is presented in Figure 1. Injuries among females ages 60-69 were the most commonly seen in the office. A breakdown of pickleball- and paddleball-related injuries and treatments is presented in Table 1. The most common anatomic location of injury was the wrist (44.7%) and the most common injury presenting to a hand surgeon was a wrist fracture (31.4%), while the most common mechanism of injury was the patient falling or diving for the ball (41.2%). For patients who underwent surgical treatment, the majority had open reduction and internal fixation (ORIF) of a wrist fracture (71.8%), and for patients treated nonoperatively, the majority were splinted (69.1%).

**Table 1:** Pickleball- and paddleball-related injuries and treatments. Fx: fracture; ORIF: open reduction and internal fixation; CTR: carpal tunnel release; TFR: trigger finger release; FDS: flexor digitorum superficialis.

Anatomic location of injury (N=217, some patients had multiple injuries)	N (%)
Wrist	97 (44.7)
Elbow	40 (18.4)
Hand	36 (16.5)
Finger	12 (5.5)
Thumb	12 (5.5)
Shoulder	9 (4.1)
Forearm	7 (3.2)
Arm	4 (1.8)
Injury description (N=204 patients)	N (%)
Wrist Fx	64 (31.4)
Unknown	62 (30.1)
Lateral/medial epicondylitis	30 (14.7)
Metacarpal Fx	17 (8.2)
Tendonitis	10 (4.9)
Other	10 (4.9)
Tenosynovitis	7 (3.4)
Trigger finger	4 (2.0)
Mechanism of injury (N=204 patients)	N (%)
Fall/dive	84 (41.2)
Playing sport	61 (30.0)
Unknown	30 (14.7)
Use/overuse	11 (5.4)
Other	10 (4.9)
Racket/paddle/ball injury	8 (3.9)
Surgical treatment (N=39 patients)	N (%)
ORIF	28 (71.8)
Distal bicep tendon repair	3 (7.7)
Closed reduction and pinning	2 (5.1)
CTR	1 (2.6)
Lateral epicondyle fasciectomy	1 (2.6)
TFR and FDS tenotomy	1 (2.6)
TFR and synovectomy	1 (2.6)
Rotator cuff repair	1 (2.6)
Tennis elbow repair	1 (2.6)
Conservative treatment (N=165 patients, majority of patients had more than one treatment)	N (%)
Orthotics/splint/brace	114 (69.1)
Therapy	73 (44.2)
Steroid injection	43 (26.1)

## Discussion

Prior literature on pickleball or paddleball injuries is primarily based on ED visits from national databases. In this study, we focused on the outpatient office presentation of these injuries to the hand and wrist surgery division of a single orthopedic practice.

An individualized chart review of 204 patients treated over eight years with pickleball or paddleball injuries allowed us to gain a more detailed understanding of these injuries and their treatments and to compare them to broader national database studies. We found that the most common injury involved the wrist due to trauma from falling. While these injuries were commonly treated with a splint or brace, a significant number of patients were treated surgically (approximately one in five). The most common procedure was ORIF of the distal radius.

The two other published reports of pickleball injuries, Forrester [8] in 2019 and Weiss et al. [7] in 2021, broadly documented national trends in these patients as they related to ED visits. Forrester [8] identified 300 pickleball injuries over five years (2013-2017). The most common injuries were sprain (28.7%) and fractures (27.7%), which occurred more frequently in the lower extremity than the upper extremity (32.0% and 25.4%, respectively). The majority of patients were over 50 years of age (90.9%) [8]. While our paper did not examine cases of lower extremity injuries, the major types of upper extremity injuries were the same including strains and fractures. However, our findings indicated that fractures (39.7%) were a more common injury in the upper extremity compared to strains (23%). Furthermore, patients with fractures, compared to strains or sprains, may be more likely to follow up in an outpatient setting with an orthopedist after the initial ED visit, which may explain why there is a higher number of fractures seen in the outpatient setting.

Weiss et al. [8] conducted a similar search identifying 429 patients. Although there was equal participation of men and women, this study found that females were more likely to be diagnosed with a fracture than males. The most common mechanism of injury was a slip/fall, and the most common location of the fracture was the wrist [7]. This finding correlated with our study, which found that the most common injury pickleball/paddleball players sustained was a wrist fracture (31%). Additionally, we also found that more females than males presented with a pickleball injury. A possible reason is that older women have decreased bone density, leading to an increased risk of fracture [9,10]. 

In a paper discussing injury considerations in pickleball, Greiner [11] predicted that sprain/strain of the lower extremity would be common due to its prevalence in tennis and the similarity in style of play requiring a greater degree of running [11]. Forrester [8] did not examine the locations of specific kinds of injuries, but considering the most common mechanism of injury was a fall or dive, improper balance and lateral movement are likely major contributors to injuries in pickleball. Furthermore, the advanced age of the average pickleball player, as senior players have less bone density, less general conditioning, and diminished motor skills compared to younger players, may be significant contributing factors to falls as the most frequent injury.

With the increasing popularity of pickleball/paddleball, there can be an expected rise in the rate of injury among such players [7]. There has also been a large increase in young players attributed to the addition of the sport to the physical education curriculum at high schools and middle schools, which would be expected to shift the demographic of patients and types of injuries [12]. It is in the best interest of orthopedic surgeons to advise players of this sport on proper techniques for injury prevention. Previous papers have recommended proper footwear to stabilize movement [11-13]. We support this recommendation considering slips/falls were the most common mechanism of injury in ours and other studies [7,8]. Vitale and Liu [12] also suggested taping the wrist to avoid aggravating chronic wrist injuries. While this may support wrist stability, it is not known whether this type of prevention will affect the quality of play or the incidence of sprain, strain, or fractures. The same author recommended that players have a light grip on the paddle as well, which is also seen in tennis as different grip positions and strengths can reduce or increase certain upper extremity injuries [12,14]. With only one arm used to swing, a light grip can be useful in preventing strains, sprains, or aggravating chronic injuries [12].

The previous papers that analyzed the incidence and the most common types of pickleball injuries had some limitations. One limitation is that the National Electronic Injury Surveillance System (NEISS) article does not capture patients treated outside the ED [7,8]. Our study adds to the current research by analyzing patients from a hand and upper extremity outpatient clinic and providing a more detailed evaluation of pickleball/paddleball players and their injuries. Additionally, the NEISS is not able to track the follow-up and definitive treatment of identified cases. Our study reviewed all patients with pickleball/paddleball injuries including follow-up care to document the most common treatments.

To our knowledge, there were no previous studies reviewing treatments of pickleball- or paddleball-related upper extremity injuries. We found that 80% of patients with more limited injuries opted for nonsurgical treatments managed by steroid injection, splinting, or therapy. The most common surgical treatment was ORIF for a distal radius fracture due to a fall or dive (72%). This aligns with other observational studies that found plate fixation was the most common surgical treatment for the same type or pattern of injury [15]. Our findings suggest that patients above the age of 65 playing pickleball or paddleball who sustain a distal radius fracture require surgery at a higher rate than the general population. A possible explanation for this trend is that pickleball and paddleball players are actively moving, contributing to a higher velocity at impact and a more serious fracture. It is assumed that those participating in pickleball/paddleball are on average younger, would have a more active lifestyle with greater functional demands, and are more likely to opt for surgical management. Additionally, several studies have demonstrated consistently good outcomes in the elderly treated conservatively with distal radius fractures, and this may be another reason for the disparity in treatment between the pickleball/paddleball players and the Medicare population cohorts [15,16]. Future studies could compare the general rate of surgery for patients sustaining a distal radius fracture in the Philadelphia area with the rate found in this study.

As seen in many retrospective reviews, one limitation of this study is only having access to pre-existing patient data. When searching for keywords, patients or physicians who did not mention pickleball or paddleball in the visit notes would be missed with our methodology. Additionally, some physicians refer to paddleball as “padel” tennis, even if they are slightly different sports. This was not known to us at the time of data collection; however, we do not think that this issue had a significant implication in our study considering that this terminology is not used in the Philadelphia area.

While we tracked treatments for each patient, we did not track treatment complications or the patients’ success in returning to play. These outcomes, as well as the incidence of injury to the dominant or nondominant hand, could be investigated in a future study. With the rapidly growing number of players, it can be anticipated that future studies will have a larger pool of patients from which to collect data and perform more extensive research on these popular sports [7,8].

## Conclusions

This retrospective case series reports on the incidence of patients with pickleball/paddleball-related injuries presenting to a hand and upper extremity surgeon in the outpatient setting. The majority of these patients sustained wrist injuries due to a fall/dive, and the majority of these sport-related injuries were treated nonoperatively. The most common surgical treatment, when required, was ORIF of the distal radius, and there was a higher surgery rate with pickleball and paddleball players who were 65 years and over. As pickleball and paddleball continue to gain popularity, hand surgeons should be aware of the types of injuries, treatments, and outcomes associated with these sports and, when possible, counsel patients accordingly to try to prevent them.
